# Measure Theoretic Entropy of Discrete Geodesic Flow on Nagao Lattice Quotient

**DOI:** 10.3390/e23010052

**Published:** 2020-12-31

**Authors:** Sanghoon Kwon

**Affiliations:** Department of Mathematical Education, Catholic Kwandong University, Gangneung 25601, Korea; skwon@cku.ac.kr

**Keywords:** measure theoretic entropy, field of formal series, diagonal action

## Abstract

The discrete geodesic flow on Nagao lattice quotient of the space of bi-infinite geodesics in regular trees can be viewed as the right diagonal action on the double quotient of $PGL_{2}\;\left(F_{q}\left(\;\left(t^{-1}\right)\;\right)\right)$ by $PGL_{2}\;\left(F_{q}\left[t\right]\right)$ and $PGL_{2}\left(F_{q}\left[\;\left[t^{-1}\right]\;\right]\right)$. We investigate the measure-theoretic entropy of the discrete geodesic flow with respect to invariant probability measures.

## 1. Introduction

Let (X,B,μ) be a probability space and let T:X→X be a measurable map. We say that *T* is measure-preserving if $\mu \left(T^{-1}A\right)=\mu \left(A\right)$ for every A∈B. In this case we say that (X,B,μ,T) is a *measure-preserving system*. To a measure-preserving system is associated a numerical invariant called measure-theoretic entropy (see Section 2 for the precise definition). Since it is preserved by measurable isomorphism, it can be used in order to distinguish special measures like Haar measure from other invariant measures.

One of the most important dynamical systems in homogeneous dynamics is the geodesic flow on the quotient $PSL\left(2,Z\right)\backslash T^{1}H$ of the unit tangent bundle $T^{1}H$ of hyperbolic plane by modular group. It is an Anosov flow on a three-dimensional non-compact manifold and has wide application on the theory of Diophantine approximation and analytic number theory. Using the Mobius transformation of PSL(2,R) on H, it may be identified with $T_{a_{t}}:X\to X$ on X=SL(2,Z)\SL(2,R)/SO(2) given by $x\mapsto xa_{t}$ with

$$a_{t}=\left(\begin{array}{cc} e^{\frac{t}{2}} & 0\\ 0 & e^{-\frac{t}{2}} \end{array}\right).$$

Unlike in the case of unipotent flow (right multiplication by one-parameter unipotent group), there is a great variety of invariant probability measures and orbit closures of $T_{a_{t}}$ on *X*. Furthermore, according to Sullivan [1], its supremum of measure theoretic entropy is equal to 1, which is the measure-theoretic entropy of the Haar measure.

Meanwhile, the discrete version of the geodesic flow is also explored by several authors ([2,3,4]). They considered the behavior of discrete geodesic flow system on and its application on Diophantine approximation over a positive characteristic field of formal series. Following these literatures, we investigate the measure-theoretic entropy of the discrete geodesic flow on positive characteristic setting in this paper. More precisely, we compute the measure-theoretic entropy of the right translation by diagonal elements on the non-compact quotient $PGL\left(2,F_{q}\left[t\right]\right)\backslash \mathrm{GT}{\;}_{q+1}$ of the space of bi-infinite geodesics ${\mathrm{GT}}_{q+1}$ of (q+1)-regular tree by modular group. It also may be viewed as a diagonal action on the positive characteristic homogeneous space $PGL_{2}\;\left(F_{q}\left[t\right]\right)\;\backslash PGL_{2}\;\left(F_{q}\left(\;\left(t^{-1}\right)\;\right)\right)\;/PGL_{2}\;\left(F_{q}\left[\;\left[t^{-1}\right]\;\right]\right)$. (See Section 3 for the action of the group on the tree.)

In the sequel, let $G=PGL_{2}\;\left(F_{q}\left(\;\left(t^{-1}\right)\;\right)\right)$, $\Gamma =PGL_{2}\;\left(F_{q}\left[t\right]\right)$, *a* be the diagonal element

$$\left[\left(\begin{array}{cc} t & 0\\ 0 & 1 \end{array}\right)\right]$$

in *G* and $T=PGL_{2}\;\left(F_{q}\left[\;\left[t^{-1}\right]\;\right]\right)$. We denote by $\phi_{a}:\Gamma \backslash G/T\to \Gamma \backslash G/T$ the right translation map given by x↦xa.

As in the real case, there are a lot of $\phi_{a}$-invariant probability measures μ on Γ\G/T. In this article, we describe these invariant probability measure μ with respect to a family of measures on $F_{q}\left(\;\left(t^{-1}\right)\;\right)$ and discuss a formula of the measure-theoretic entropy $h_{\mu }\left(\phi_{a}\right)$ of $\phi_{a}$ with respect to μ. We give the main theorem of the paper.

**Theorem** **1.** *Let $\phi_{a}:\Gamma \backslash G/T\to \Gamma \backslash G/T$ be the right translation map given as above. For each i≥1, let $E_{i}=\left\{\alpha \in F_{q}\left(\;\left(t^{-1}\right)\;\right):\deg\left(\alpha \right)=i\right\}$. If μ is the $\phi_{a}$-invariant measure on Γ\G/T, then there are measures $\mu_{i}$ on $E_{i}$ and a function $f_{\mu }:\left\{\alpha \in F_{q}\left(\;\left(t^{-1}\right)\;\right):\deg\left(\alpha \right)\geq 1\right\}\to R$ such that the following holds.*

1. $\sum_{i=1}^{\infty }2i\mu_{i}\left(E_{i}\right)=1.$
2. $h_{\mu }\left(\phi_{a}\right)=\sum_{i=1}^{\infty }\int_{E_{i}}f_{\mu }\left(\alpha \right)d\mu_{i}\left(\alpha \right).$

It is well known that the Haar measure (the unique *G*-invariant probability measure) *m* is the measure of maximal entropy for $\phi_{a}$ on Γ\G/T (see Reference [5]). For the Haar measure *m* on Γ\G/T, we can explicitly compute $m_{i}$ and $f_{m}$ of the Theorem 1. Namely, we have (see Section 5)

$$m_{i}\left(E_{i}\right)=\frac{{\left(q-1\right)}^{2}}{2}q^{-i-1}\;\mathrm{and}\;f_{m}\left(\alpha \right)=2\deg(\alpha )\log q.$$

From the above description, we achieve the measure-theoretic entropy of $\phi_{a}$ with respect to *m*.

**Corollary** **1.** *Let $\phi_{a}:\Gamma \backslash G/T\to \Gamma \backslash G/T$ be as above. Then, we have*

$$\underset{\mu }{\sup}\;h_{\mu }\left(\phi_{a}\right)=\log q$$

*and the measure of maximal entropy is the unique G-invariant probability measure. Here, supremum runs over the set of $\phi_{a}$-invariant probability measures on Γ\G/T.*

This article is organized as follows. In Section 2, we review elementary definition and some properties of measure-theoretic entropy in view of ergodic theory and dynamical systems. We study some arithmetic and geometry of $F_{q}\left(\;\left(t^{-1}\right)\;\right)$ in Section 3. There we mainly present the brief theory of simple continued fraction of $F_{q}\left(\;\left(t^{-1}\right)\;\right)$ and describe the Bruhat-Tits tree of $PGL\;\left(2,F_{q}\left(\;\left(t^{-1}\right)\;\right)\right)$. In Section 4, we investigate the dynamical system $\left(\Gamma \backslash G/T,B,\phi_{a}\right)$, describing $\phi_{a}$ on the Γ-quotient of the space of parametrized bi-infinite geodesics over the Bruhat-Tits tree of *G* by a suspension map of a shift map. Finally, we prove Theorem 1 and Corollary 1 in Section 5.

## 2. Preliminaries on Entropy

We start with the summary of some elementary definitions and properties of measure-theoretic entropy, mainly following Reference [6]. We review the entopy of a partition and that of a measure-preserving transformation.

### 2.1. Entropy of a Partition

Let us begin with some definitions. A *probability vector* $\left(p_{1},p_{2},p_{3},\ldots \right)$ is a vector with $0\leq p_{i}\leq 1$ for each i≥1 and $\underset{i=1}{\sum^{\infty }}p_{i}=1$. Given a probability vector $p=(p_{1},p_{2},p_{3},\ldots )$, let

$$H\left(p\right)=H\left(p_{1},p_{2},p_{3},\ldots \right)=-\sum_{i=1}^{\infty }p_{i}\log p_{i}.$$

Here, 0log0 is defined to be 0. A *partition* of a probability space (X,B,μ) is a finite or countably infinite collection of disjoint measurable subsets $\xi =\{A_{1},A_{2},\ldots \}$ of *X* whose union is *X*. The *entropy* $H_{\mu }\left(\xi \right)$ of a partition $\xi ={\left\{A_{i}\right\}}_{i\in I}$ with respect to a measure μ is defined by

$$H_{\mu }\left(\xi \right)=H\left(\mu \left(A_{1}\right),\mu \left(A_{2}\right),\ldots \right)=-\sum_{i\in I}\mu \left(A_{i}\right)\log\mu \left(A_{i}\right)\in \left[0,\infty \right].$$

If ξ and η are partitions, then the *conditional entropy* of ξ given η is defined to be

$$H_{\mu }\left(\xi |\eta \right)=\sum_{j=1}^{\infty }\mu \left(B_{j}\right)H\;\left(\frac{\mu (A_{1}\cap B_{j})}{\mu (B_{j})},\frac{\mu (A_{1}\cap B_{j})}{\mu (B_{j})},\ldots \right),$$

which may be viewed as a weighted average of entropies of the partition ξ conditioned on individual atoms $B_{j}\in \eta$.

The *information function* of a partition ξ is defined by

$$I_{\mu }\left(\xi \right)\left(x\right)=-\log\mu \left({\left[x\right]}_{\xi }\right)$$

where ${\left[x\right]}_{\xi }\in \xi$ is the partition element with $x\in {\left[x\right]}_{\xi }$. If η is another partition, then the *conditional information function* of ξ given η is defined by

$$I_{\mu }\left(\xi |\eta \right)\left(x\right)=-\log\frac{\mu ({\left[x\right]}_{\xi \vee \eta })}{\mu ({\left[x\right]}_{\eta })}.$$

The following proposition summarizes some important properties of entropy and information function.

**Proposition** **1** (Lemma 1.7 and Lemma 1.12 of Reference [6]). *Let (X,B,μ,T) be a measure-preserving system and let ξ,η be partitions. Then, we have*

1. *(Integration)*
  $$H_{\mu }\left(\xi \right)=\int_{X}I_{\mu }\left(\xi \right)d\mu \;and\;H_{\mu }\left(\xi |\eta \right)=\int_{X}I_{\mu }\left(\xi |\eta \right)d\mu$$
2. *(Additivity)*
  $$H_{\mu }\left(\xi \vee \eta \right)=H_{\mu }\left(\eta \right)+H_{\mu }\left(\xi |\eta \right)\;and\;I_{\mu }\left(\xi \vee \eta \right)=I_{\mu }\left(\eta \right)+I_{\mu }\left(\xi |\eta \right)$$
3. *(Invariance)*
  $$H_{\mu }\left(\xi |\eta \right)=H_{\mu }\left(T^{-1}\xi |T^{-1}\eta \right)\;and\;I_{\mu }\left(\xi |\eta \right)\circ T=I_{\mu }\left(T^{-1}\xi |T^{-1}\eta \right).$$

**Proof.**  These follows directly from the definitions. For convenience of the reader, we prove the third statement of the proposition. Since $T^{-1}{\left[Tx\right]}_{\eta }={\left[x\right]}_{T^{-1}\eta }$ for all x∈X, we have

$$\begin{array}{c} I_{\mu }\left(\xi |\eta \right)\left(Tx\right)=-\log\frac{\mu ({\left[Tx\right]}_{\xi }\cap {\left[Tx\right]}_{\eta })}{\mu ({\left[Tx\right]}_{\eta })}=-\log\frac{\mu ({\left[x\right]}_{T^{-1}\xi }\cap {\left[x\right]}_{T^{-1}\eta })}{\mu ({\left[x\right]}_{T^{-1}\eta })}=I_{\mu }\left(T^{-1}\xi |T^{-1}\eta \right)\left(x\right), \end{array}$$

which completes the proof. □

### 2.2. Entropy of a Measure-Preserving Transformation

The third observation of Proposition 1 enables us to define the notion of entropy of a measure-preserving transformation (rather than a partition). We note that the sequence $\left(a_{n}\right)$ defined by

$$a_{n}=H_{\mu }\left(\xi \vee T^{-1}\xi \vee \cdots \vee T^{-(n-1)}\xi \right)$$

is sub-additive. Hence, by Fekete’s lemma (Lemma 1.13 of Reference [6]), it follows that $\lim_{n\to \infty }a_{n}$ exists. The *entropy of* *T* *with respect to* ξ is defined by

$$h_{\mu }\left(T,\xi \right)=\lim_{n\to \infty }\frac{1}{n}H_{\mu }\left(\underset{i=1}{\overset{n-1}{\vee }}T^{-i}\xi \right)$$

and the *entropy* of *T* is defined by

$$h_{\mu }\left(T\right)=\underset{\xi :H_{\mu }\left(\xi \right)<\infty }{\sup}h_{\mu }\left(T,\xi \right).$$

If ξ is a countable partition with finite entropy, then

$$h_{\mu }\left(T,\xi \right)=\lim_{n\to \infty }H_{\mu }\left(\xi |\underset{i=1}{\overset{n}{\vee }}T^{-i}\xi \right).$$

Hence, the definition involves a supremum over the set of all finite partitions. The next theorem gives a sufficient condition on a partition to allow to work with a single partition.

**Theorem** **2** (Kolmogorov-Sinai, Theorem 1.21 of Reference [6]). *If T is invertible and ξ is a partition with finite entropy that is a generator under T in the sense that*

$$\underset{n=-\infty }{\overset{\infty }{\vee }}T^{-n}\xi =B,$$

*then $h_{\mu }\left(T\right)=h_{\mu }\left(T,\xi \right)$.*

Thus, it transfers the difficulty inherent in computing measure-theoretic entropy to the problem of finding a generating partition.

## 3. Continued Fraction of $F_{q}\left(\;(t^{-1})\;\right)$ and the Tree of $PGL_{2}$

In this section, we discuss arithmetic and geometry of a field of formal series $F_{q}\left(\;\left(t^{-1}\right)\;\right)$ over a finite field $F_{q}$. In particular, we review simple continued fraction expansion of $F_{q}\left(\;\left(t^{-1}\right)\;\right)$ and the Bruhat-Tits tree of $PGL\;\left(2,F_{q}\left(\;\left(t^{-1}\right)\;\right)\right)$. We refer to Reference [7] and Reference [4] for more details of the theory of continued fraction of a field of formal series.

### 3.1. Continued Fraction of a Field of Formal Series

Given an arbitrary field F with an absolute value |·|, we define the finite simple continued fraction $\left[a_{0};a_{1},\ldots ,a_{n}\right]$ as

$$\left[a_{0};a_{1},\ldots ,a_{n}\right]=a_{0}+\frac{1}{a_{1}+\frac{1}{\ddots +\frac{1}{a_{n}}}}\in F$$

for $a_{0}\in F$ and $a_{1},\ldots ,a_{n}\in F-\left\{0\right\}$. We define the *infinite simple continued fraction* $\left[a_{0};a_{1},\ldots \right]$, if exists, by

$$\left[a_{0};a_{1},a_{2},\ldots \right]=\lim_{n\to \infty }\left[a_{0};a_{1},\ldots ,a_{n}\right]$$

where the limit is taken with respect to the absolute value |·|.

Let K be the field $F_{q}\left(\;\left(t^{-1}\right)\;\right)$ of Laurent series in $t^{-1}$ over a finite field $F_{q}$ and Z be the subring $F_{q}\left[t\right]$, of polynomials in *t* over $F_{q}$, of K. Given an element $\alpha =\sum_{i=n}^{-\infty }a_{i}t^{i}$ of K with $a_{n}\neq 0$, let us define

$$\begin{array}{cc} \deg(\alpha )=n,\;[\alpha ] & =a_{0}+a_{1}t+\cdots +a_{n}t^{n},\;\left\{\alpha \right\}=a_{-1}t^{-1}+a_{-2}t^{-2}+\cdots \end{array}$$

the degree, the polynomial part and fractional part of α, respectively. Then, K is a normed field with the associated absolute value given by

$$\left|\frac{\alpha }{\beta }\right|=q^{\deg(\alpha )-\deg(\beta )},\;\left|0\right|=0.$$

We further denote by O the local ring $F_{q}\left[\;\left[t^{-1}\right]\;\right]$ of K which consists of power series in $t^{-1}$ over $F_{q}$. More precisely, let

O={α∈K: deg(α)≤0}.

Contrary to the usual absolute value on Q, the norm |·| on K is non-Archimedean, that is,

|α−β|≤max{|α|,|β|}

holds for every α,β∈K and in particular equality holds if |α|≠|β|.

While there is no general algorithm to compute the sum, difference or product of continued fractions, we state a useful lemma on an absolute value of difference of two continued fractions.

**Lemma** **1** (Lemma 1.2.21 of Reference [7]). *For*

$$\alpha =\left[a_{0};a_{1},a_{2},\ldots \right]\;and\;\beta =\left[b_{0};b_{1},b_{2},\ldots \right]$$

*with α≠β, let i be the integer such that $a_{n}=b_{n}$ for n=0,1,…,i−1 and $a_{i}\neq b_{i}$. If i=0, then $\left|\alpha -\beta |=\right|a_{0}-b_{0}|$. If i≥1, then*

$$\left|\alpha -\beta \right|=\frac{|a_{i}-b_{i}|}{q^{2d_{i}}\left|a_{i}b_{i}\right|}$$

*where $d_{i}=\deg\left(a_{1}\right)+\cdots +\deg\left(a_{i-1}\right)$.*

The non-Archimedean property of the norm |·| on K yields that $a_{0}=b_{0},\ldots ,a_{i}=b_{i}$ if and only if $|\alpha -\beta |<q^{-2d_{i}}$ with the above notation. We conclude that the infinite simple continued fraction expansion of a Laurent series is always unique.

### 3.2. Tree of $PGL_{2}$

We recall the notion of *Bruhat-Tits tree* T of *G* in this subsection. See also Reference [5] for the detail. Let *W* the maximal compact subgroup $PGL_{2}\left(O\right)$ of *G*. The vertices of T are defined to be the elements of G/W. We note that right multiplication of elements in *W* corresponds to an iteration of elementary O-column operations. Let us recall that there are three types of elementary O-column operations.

1. A column within the matrix can be switched with another column.
2. Each column can be multiplied by an invertible element of O (hence by a non-zero element of $F_{q}$).
3. A column can be replaced by the sum of that column and a O-multiple of another column.

Using these three types of operations, we can understand every vertex of T as

$$\left[\left(\begin{array}{cc} t^{n} & f(t)\\ 0 & 1 \end{array}\right)\right]W$$

for some integer *n* (may be negative) and a rational function $f\left(t\right)\in t^{n+1}Z$. Let

$$\pi_{n}:t^{n}Z\to t^{n+1}Z,\;\pi_{n}\left(a_{n}t^{n}+a_{n+1}t^{n+1}+\cdots \right)=a_{n+1}t^{n+1}+\cdots$$

be the projection map which forgets the $t^{n}$ term. Two vertices

$$\left[\left(\begin{array}{cc} t^{n_{1}} & f_{1}\left(t\right)\\ 0 & 1 \end{array}\right)\right]W\mathrm{and}\;\left[\left(\begin{array}{cc} t^{n_{2}} & f_{2}\left(t\right)\\ 0 & 1 \end{array}\right)\right]W$$

are defined to be adjacent to each other (there is an edge between two vertices) if and only if $|n_{1}-n_{2}|=1$ and $f_{1}$ and $f_{2}$ satisfy

$$\left\{\begin{array}{cc} f_{2}\left(t\right)=\pi_{n_{2}}\left(f_{1}\left(t\right)\right), & \mathrm{if}\;n_{2}=n_{1}+1\\ f_{2}\left(t\right)=f_{1}\left(t\right)+at^{n_{1}}, & \mathrm{if}\;n_{2}=n_{1}-1 \end{array}\right.$$

for some $a\in F_{q}$. It follows that the degree (the number of edges attached to the vertex) of each vertex of T is equal to q+1. We also note that the visual boundary $\partial_{\infty }\;T$ at infinity of T can be identified with $P^{1}\left(K\right)=K\cup \left\{\infty \right\}$ (cf. Section 2 of Reference [8]). Let $\partial_{\infty }\;T_{\mathrm{dist}}^{3}$ be the set

$$\left\{\left(\omega_{1},\omega_{2},\omega_{3}\right)\in {\left(\partial_{\infty }\;T\right)}^{3}:\omega_{i}\neq \omega_{j}\mathrm{for}1\leq i\neq j\leq 3\right\}$$

of distinct ordered triple points in $\partial_{\infty }\;T$. Since two by two projective general linear group PGL(2,F) over a field *F* acts simply transitively on ${\left(P^{1}\left(F\right)\right)}_{\mathrm{dist}}^{3}$ by Möbius transformation

$$\left[\begin{array}{cc} a & b\\ c & d \end{array}\right]\;\cdot \;\omega =\frac{a\omega +b}{c\omega +d},$$

we have a bijection $\Phi :\;G\to \partial_{\infty }\;T_{\mathrm{dist}}^{3}≃P^{1}{\left(K\right)}_{\mathrm{dist}}^{3}$ given by

Φ(g)=g·(0,1,∞).

Let us finish this section with introducing notation for special vertices of T. Let $x_{i}$ be the vertex of T defined by

$$x_{i}=\left[\left(\begin{array}{cc} t^{i} & 0\\ 0 & 1 \end{array}\right)\right]W.$$

**Figure 1:**
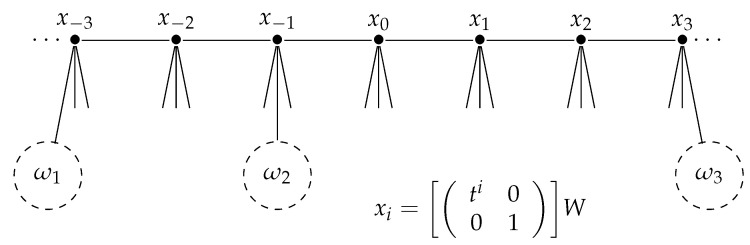
Example: $\deg(\omega_{1})=-3$, $\deg(\omega_{2})=-1$ and $\deg(\omega_{3})=3$.

Then, the sequence ${\left(x_{i}\right)}_{i=-\infty }^{\infty }$ forms a bi-infinite parametrized geodesic on T, which we call the *standard geodesic* of T. See Figure 1 which describes the vertices $x_{i}$ of T and an example of ordered triple points $\left(\omega_{1},\omega_{2},\omega_{3}\right)$.

## 4. Quotient by Nagao Lattice and a Partition of Γ\G/T

In this section, we look into the diagonal action $\phi_{a}$ on Γ\G/T in details. We show that the right translation map by diagonal elements on Γ\G/T can be viewed as a suspension map of a shift space. From this, we may find a generating partition of $\left(\Gamma \backslash G/T,\phi_{a}\right)$ with Borel σ-algebra, which is useful when we compute the measure-theoretic entropy. The main ingredient of the proof is the uniqueness of infinite simple continued fraction expansion on K.

### 4.1. Describing $\phi_{a}$ on Γ\G/T as a Suspension System

The group Γ acts on the set of vertices of T by γ·gW=γgW. Let us recall that there are three types of elementary Z-row operations.

1. A row within the matrix can be switched with another row.
2. Each row can be multiplied by an invertible element of Z (hence by a non-zero element of $F_{q}$).
3. A row can be replaced by the sum of that row and a Z-multiple of another row.

In a similar way as in the previous section, we note that left multiplication of elements in Γ corresponds to an iteration of elementary Z-row operations. Applying these operations, we may write *G* as a union of double coset

$$G=\underset{i=0}{\overset{\infty }{\cup }}\Gamma \left[\left(\begin{array}{cc} t^{i} & 0\\ 0 & 1 \end{array}\right)\right]W.$$

**Figure 2:**
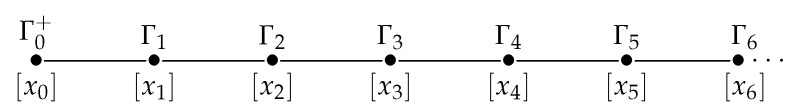
Nagao ray of index *q*.

Therefore, the quotient graph Γ\T is a ray (see Figure 2) with vertices

$$\left[x_{i}\right]=\Gamma \left[\left(\begin{array}{cc} t^{i} & 0\\ 0 & 1 \end{array}\right)\right]W,\;i=0,1,2,\ldots$$

whose stabilizer in Γ is given by

$${\mathrm{Stab}}_{\Gamma }\left(x_{0}\right)=\Gamma_{0}^{+}=PGL\left(2,F_{q}\left[t\right]\right)$$

and

$${\mathrm{Stab}}_{\Gamma }\left(x_{i}\right)=\Gamma_{i}=\left\{\left(\begin{array}{cc} a & b\\ 0 & d \end{array}\right)\;\in \;\Gamma :\;\deg\left(b\right)\leq i\right\}.$$

Let $\phi_{a}:\;\Gamma \backslash G/T\to \Gamma \backslash G/T$ be the map given by $\phi_{a}\left(x\right)=xa$. In Section 3 of Reference [8], the author identified Γ\G/T with

$$\{\left(\omega_{1},\omega_{2},k\right):\omega_{1}\in t^{-1}O,\omega_{2}\in K-O,\deg\left(\omega_{1}\right)\leq k<\deg\left(\omega_{2}\right)\}.$$

Slightly modifying the argument, we may also identify Γ\G/T with

$$F=\{\left(\omega_{1},\omega_{2},k\right):\omega_{1}\in t^{-1}O,\omega_{2}\in K-O,0\leq k<2\deg\left(\omega_{2}\right)\}$$

and the map $\phi_{a}$ is equivariant with the map ϕ:F→F given by

$$\begin{array}{cc} \phi (\omega_{1},\omega_{2},k)= & \left\{\begin{array}{cc} \left(\omega_{1},\omega_{2},k+1\right) & \mathrm{if}\;0\leq k<2\deg(\omega_{2})-1\\ \left(\frac{1}{\omega_{1}-\left[\omega_{2}\right]},\frac{1}{\omega_{2}-\left[\omega_{2}\right]},0\right) & \mathrm{if}\;k=2\deg(\omega_{2})-1. \end{array}\right. \end{array}$$

**Figure 3:**
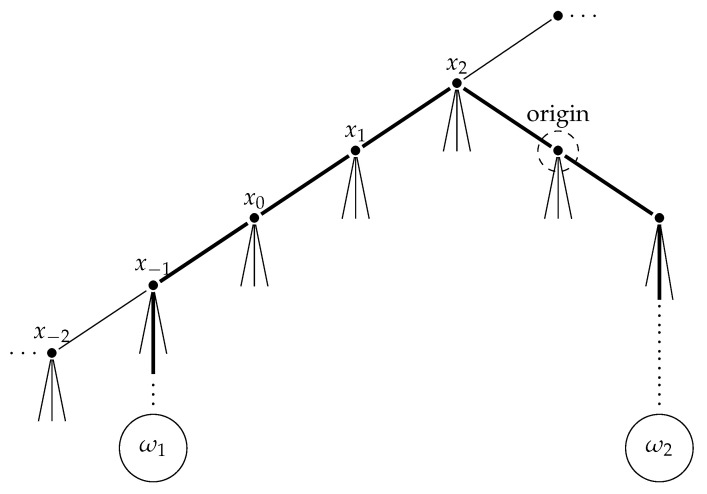
$\left(\omega_{1},\omega_{2},k\right)$ with $\deg(\omega_{1})=-1$, $\deg(\omega_{2})=2$, k=3.

See Figure 3 for the case of $\left(\omega_{1},\omega_{2},3\right)$ with $\deg(\omega_{1})=-1$ and $\deg(\omega_{2})=2$.

In other words, we have the following commutative diagram.

$$\begin{array}{ccc} \Gamma \backslash G/T & \overset{\phi_{a\;}}{⟶} & \Gamma \backslash G/T.\\ \Phi ↓ & & \Phi ↓\\ F & \overset{\;\phi \;}{⟶} & F \end{array}$$

This enables us to consider the system $\left(\Gamma \backslash G/T,\phi_{a}\right)$ as a suspension map on $t^{-1}O\times \left(K-O\right)$ with the roof function $r\left(\omega_{1},\omega_{2}\right)=2\deg\left(\omega_{2}\right)$.

### 4.2. Entropy Generating Partition of Γ\G/T

In this subsection, we give a generating partition of $\left(\Gamma \backslash G/T,\phi_{a}\right)$. Let $\left[0;a_{1},a_{2},\ldots \right]$ be the simple continued fraction of $\omega_{1}$. If $\omega_{1}$ is rational and has a finite continued fraction $\left[0;a_{1},\ldots ,a_{n}\right]$, then we write as

$$\omega_{1}=\left[0;a_{1},\ldots ,a_{n},\infty ,\infty ,\ldots \right].$$

Hence, we may assume that the continued fraction $\left[0;a_{1},a_{2},\ldots \right]$ is always infinite. Similarly, let $\left[b_{0};b_{1},b_{2},\ldots \right]$ be the infinite simple continued fraction of $\omega_{2}$.

In order to explain the basis of F, let us introduce a notation. Let

$$E_{\alpha ,\beta ,k}^{n_{1},n_{2}}=\left(\alpha +t^{-n_{1}}O\right)\times \left(\beta +t^{-n_{2}}O\right)\times \left\{k\right\}.$$

**Figure 4:**
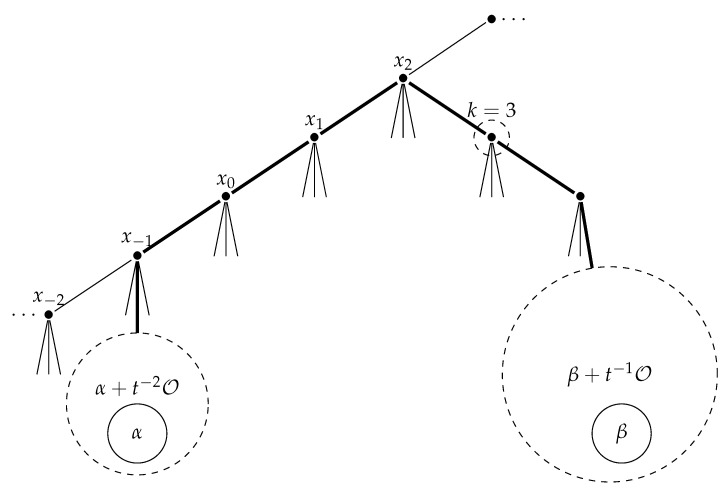
$E_{\alpha ,\beta ,k}^{2,1}$ with deg(α)=−1, deg(β)=2, k=3.

The collection

$$\left\{E_{\alpha ,\beta ,k}^{n_{1},n_{2}}:\alpha \in t^{-1}O,\beta \in K-O,n_{1}\geq 1,n_{2}\geq 1,0\leq k<2\deg\left(\beta \right)\right\}$$

of subsets of

$$\left\{\left(\omega_{1},\omega_{2},k\right):\omega_{1}\in t^{-1}O,\omega_{2}\in K-O,0\leq k<2\deg\left(\omega_{2}\right)\right\}$$

forms a basis for topology of F. See Figure 4 which describes $E_{\alpha ,\beta ,3}^{2,1}$ with deg(α)=−1 and deg(β)=2.

Let $L=F_{q}\left[t\right]\cup \left\{\infty \right\}-\left\{F_{q}\right\}$. Using the infinite simple continued fraction of K, we may write arbitrary elment $\left(\omega_{1},\omega_{2}\right)$ of $t^{-1}O\times \left(K-O\right)$ as

$$\left(\omega_{1},\omega_{2}\right)=\left(\ldots ,a_{2},a_{1},\;\;\;\underset{\mathrm{origin}}{\underset{↑}{\underline{b_{0}}}}\;\;\;,b_{1},b_{2},\ldots \right)\in L^{Z}$$

for

$$\omega_{1}=\left[0;a_{1},a_{2},\ldots \right]\;\mathrm{and}\;\omega_{2}=\left[b_{0};b_{1},b_{2},\ldots \right].$$

Let $\left[a_{n},\ldots ,a_{1},\underline{b_{0}},b_{1},\ldots ,b_{m}\right]$ the *cylindrical set* defined by

$$\left\{\left(c_{i}\right)\in L^{Z}:c_{-i}=a_{i}\mathrm{for}1\leq i\leq n,c_{j}=b_{j}\mathrm{for}0\leq j\leq m\right\}$$

and hence we may again identify F with a subset of $L^{Z}\times Z_{\geq 0}$. Let ξ be the partition of Γ\G/T for which the ξ-atom ${\left[x\right]}_{\xi }$ of

$$x=(\left(\ldots ,a_{n},\ldots ,a_{1},\underline{b_{0}},b_{1},\ldots ,b_{m},\ldots \right),k)$$

is given by ${\left[x\right]}_{\xi }=\left(\left[\underline{b_{0}}\right],k\right).$ Then

$$\underset{i=1}{\overset{n}{\vee }}\phi_{a}^{-i}\xi =\left\{\left(\left[\underline{b_{0}},b_{1},\ldots ,b_{j}\right],k\right):\begin{array}{c} 0\leq k<2\deg(b_{0})\\ 2(\deg\left(b_{1}\right)+\cdots +\deg\left(b_{j}\right))\leq n \end{array}\right\}.$$

**Lemma** **2.** *The partition ξ is a* generator *of $\left(\Gamma \backslash G/T,\phi_{a},B\right)$ for the Borel σ-algebra B of Γ\G in the sense that*

$$\underset{j=-(n-1)}{\overset{n-1}{\vee }}\phi_{a}^{-j}\left(\xi \right)\to B\mathrm{as}n\to \infty .$$

**Proof.**  This follows from the uniqueness of the simple continued fraction expansion of K due to Lemma 1. □

## 5. Invariant Probability Measures and Entropy

In this section, we prove Theorem 1 and Corollary 1.

### 5.1. Description of $\mu_{i}$ and $f_{\mu }$

Let us prove the first statement of Theorem 1. We may characterize the $\phi_{a}$-invariant probability measures on Γ\G/T. Note that

$$\mu \left(\left[c_{n_{1}},\ldots ,c_{1},\underline{b_{0}},b_{1},\ldots ,b_{n_{2}}\right],0\right)=\mu \left(\left[c_{n_{1}},\ldots ,c_{1},\underline{b_{0}},b_{1},\ldots ,b_{n_{2}}\right],k\right)$$

for any *k* and hence we get

$$\mu \left(\left[c_{n_{1}},\ldots ,c_{1},\underline{b_{0}},b_{1},\ldots ,b_{n_{2}}\right],0\right)=\mu \left(\left[c_{n_{1}},\ldots ,\underline{c_{1}},b_{0},b_{1},\ldots ,b_{n_{2}}\right],0\right).$$

By Lemma 1, we have

$$\left[a_{r},\ldots ,a_{1},\underline{b_{0}},b_{1},\ldots ,b_{s}\right]=\left(\alpha +t^{-2\sum \deg(a_{i})-1}O\right)\times \left(\beta +t^{-2\sum \deg(b_{j})-1}O\right).$$

Since every open basis $E_{\alpha ,\beta ,k}^{n_{1},n_{2}}$ of *F* is a union of cylindrical sets, a $\phi_{a}$-invariant probability measure μ is determined by the value of

$$\mu (\left[\underline{b_{0}},b_{1},\ldots ,b_{s}\right],0).$$

We may consider F as a disjoint union

$$F=\underset{i=1}{\overset{\infty }{\cup }}\underset{k=0}{\overset{2i-1}{\cup }}\left\{\left(\omega_{1},\omega_{2},k\right):\;\deg\left(\omega_{2}\right)=i\right\}.$$

Let $U_{b_{0},b_{1},\ldots ,b_{n}}$ be the open subset of K defined by

$$\left\{\alpha \in K:\;\alpha =\left[a_{0};a_{1},\ldots ,a_{n},\ldots \right],a_{i}=b_{i}\mathrm{for}0\leq i\leq n\right\}.$$

Let $\mu_{i}$ be the measure on $E_{i}$ given by $\mu_{i}\left(U_{b_{0},b_{1},\ldots ,b_{n}}\right)=\mu \left(\left[\underline{b_{0}},b_{1},\ldots ,b_{n}\right],0\right)$ for $\deg(b_{0})=i$. 

Since

$$F=\underset{c\in L}{\cup }\underset{k=0}{\overset{2\deg(c)-1}{\cup }}\left(\left[\underline{c}\right],k\right)$$

and μ(F)=1, we have

(1) $$\sum_{i=1}^{\infty }2i\mu_{i}\left(E_{i}\right)=1.$$

**Remark** **1.** *In fact, the converse also holds. Given the measure $\mu_{i}$ on $E_{i}$ satisfying the Condition 1, let μ be the measure on F defined by*

$$\mu \left(\left[c_{n},\ldots ,c_{1},\underline{b_{0}},b_{1},\ldots ,b_{m}\right],k\right)=\mu_{\deg(c_{n})}\left(U_{c_{n},\ldots ,b_{0},b_{1},\ldots ,b_{m}}\right)$$

*for any $c_{i},b_{j}\in L$ and $0\leq k<2\deg(b_{0})$. Then, μ is a $\phi_{a}$-invariant probability measure on F.*

**Lemma** **3.** *The G-invariant probability measure m on Γ\G/T is given by*

$$m\left(E_{\alpha ,\beta ,k}^{n_{1},n_{2}}\right)=\frac{q-1}{2q^{n_{1}+n_{2}+2\deg\left(\beta \right)-1}}.$$

**Proof.**  We note that there are $\left(q-1\right)q^{i}$ polynomials of degree *i*. Since

$$F=\underset{b\in L}{\cup }\underset{k=0}{\overset{2\deg(b)-1}{\cup }}E_{0,b,k}^{1,1}$$

and

$$\sum_{b\in L}\frac{2(q-1)\deg(b)}{2q^{2\deg(b)+1}}=\sum_{i=1}^{\infty }\frac{i{\left(q-1\right)}^{2}}{q^{i+1}}=1,$$

the above definition implies that ∥m∥=1. We also note that $gx_{0}=x_{0}$ for g∈W. From the definition, *m* is invariant under *W*. Indeed,

$$m\left(E_{\alpha ,\beta ,k}^{n_{1},n_{2}}\right)=m\left(E_{\alpha^{\prime },\beta^{\prime },k}^{n_{1},n_{2}}\right)$$

for all $\alpha ,\alpha^{\prime }\in t^{-1}O$ and $\beta ,\beta^{\prime }\in K-O$ with $\deg\left(\beta \right)=\deg\left(\beta^{\prime }\right)$, so the measures are allocated with equal probability at each branch point. By Cartan decomposition $G=WA^{+}W$, a $\phi_{a}$-invariant measure is *G*-invariant if and only if it is *W*-invariant. Thus, the measure *m* is invariant under *G*. □

It also can be characterized by the value of cylindrical sets. Namely,

$$m\left(\left[c_{n},\ldots ,\underline{b_{0}},\ldots ,b_{n^{\prime }}\right],k\right)=\frac{q-1}{2}q^{-2\deg\left(c_{n}\right)-\cdots -2\deg\left(c_{1}\right)-2\deg\left(b_{0}\right)-\cdots -2\deg\left(b_{n^{\prime }}\right)-1}.$$

Let us define

$$f_{\mu }\left(\beta \right)=-\lim_{t\to \infty }\log\frac{\mu (\left[\underline{b_{0}},b_{1},\ldots ,b_{t}\right],0)}{\mu (\left[\underline{b_{1}},\ldots ,b_{t}\right],0)}.$$

**Example** **1.** *We note that there are $\left(q-1\right)q^{i}$ polynomials of degree i. From the definition of m in Lemma 3, it follows that*

$$m_{i}\left(E_{i}\right)=\frac{{\left(q-1\right)}^{2}}{2}q^{-i-1}\;and\;f_{m}\left(\alpha \right)=2\deg(\alpha )\log q.$$

### 5.2. Entropy of $\phi_{a}$ with Respect to μ and m

Now we prove the second part of Theorem 1. Let $A=\underset{n=0}{\overset{\infty }{\vee }}\phi_{a}^{-n}\xi$ be the future conditioning partition of Γ\G/T. We note that ξ is a generator of $\left(\Gamma \backslash G/T,\phi_{a},B\right)$ from Lemma 2. Since ${\left[x\right]}_{A}\neq {\left[x\right]}_{\phi_{a}^{-1}A}$ if only if $k=2\deg(b_{0})-1$, the measure-theoretic entropy of $\phi_{a}$ with respect to μ is given by

$$\begin{array}{cc} h_{\mu }\left(\phi_{a},\xi \right)= & \lim_{n\to \infty }H_{\mu }\left(\xi |\underset{i=1}{\overset{n}{\vee }}\phi_{a}^{-i}\xi \right)\\ = & \int_{\Gamma \backslash G/T}-\log\mu_{x}^{\phi_{a}^{-1}\;A}\left({\left[x\right]}_{A}\right)d\mu \left(x\right)\\ = & -\int_{F}\lim_{t\to \infty }\log\frac{\mu (\left[\underline{b_{0}},b_{1},\ldots ,b_{t}\right],2\deg\left(b_{0}\right)-1)}{\mu (\left[\underline{b_{1}},\ldots ,b_{t}\right],0)}d\mu \left(x\right)\\ = & \sum_{i=1}^{\infty }\int_{E_{i}}f_{\mu }\left(\alpha \right)d\mu_{i}\left(\alpha \right). \end{array}$$

**Corollary** **2.** *Let $\phi_{a}:\Gamma \backslash G/T\to \Gamma \backslash G/T$ be the map x↦xa and m be the unique G-invariant probability measure on Γ\G/T. Then, $h_{m}\left(\phi_{a}\right)=\log q$.*

**Proof.**  Since $f_{m}\left(\alpha \right)$ is constant (equal to $\log q^{2i}$) on each $E_{i}$, we have

$$h_{m}\left(\phi_{a}\right)=\sum_{i=1}^{\infty }\frac{i{\left(q-1\right)}^{2}}{q^{i+1}}\log q=\log q$$

from the above entropy formula. □

## 6. Discussion

From the above theorem, we may distinguish the Haar measure with other $\phi_{a}$-invariant probability measures. It would be very interesting to discuss the effective uniqueness of the maximal measure *m*. Namely, we would like to answer to the following question: For a compactly supported locally constant function *f* on $PGL_{2}\;\left(F_{q}\left[t\right]\right)\;\backslash PGL_{2}\;\left(F_{q}\left(\;\left(t^{-1}\right)\;\right)\right)\;/$$PGL_{2}\;\left(F_{q}\left[\;\left[t^{-1}\right]\;\right]\right)$, is |m(f)−μ(f)| is essentially bounded by $|h_{m}\left(\phi_{a}\right)-h_{\mu }\left(\phi_{a}\right)|$?

This type of question can be answered via achieving ‘Einsiedler inequality’. It is known for a shift of finite type [9], diagonal action on *p*-adic and *S*-arithmetic homogeneous spaces ([10,11]). In the positive characteristic setting, the main difficulty is that the associated countable Markov shift does not have the ‘big images and preimages’ (BIP) property.

## 7. Conclusions

Measure-theoretic entropy is a numerical invariant associated to a measure-preserving system. It is preserved by measurable isomorphism, and hence it can be used in order to distinguish special measures from other invariant measures. Motivated by the case of geodesic flow on modular surface PSL(2,Z)\H, we addressed a positive characteristic homogeneous space.

We investigated arbitrary invariant probability measures of the discrete geodesic flow $\phi_{a}:x\mapsto xa$ on $PGL_{2}\;\left(F_{q}\left[t\right]\right)\;\backslash PGL_{2}\;\left(F_{q}\left(\;\left(t^{-1}\right)\;\right)\right)\;/PGL_{2}\;\left(F_{q}\left[\;\left[t^{-1}\right]\;\right]\right)$. Especially, we interpreted these invariant probability measures μ with respect to a family of measures on a field $F_{q}\left(\;\left(t^{-1}\right)\;\right)$ of formal series. The formula of the mesure-theoretic entropy with respect to general $\phi_{a}$-invariant measure on $PGL_{2}\;\left(F_{q}\left[t\right]\right)\;\backslash PGL_{2}\;\left(F_{q}\left(\;\left(t^{-1}\right)\;\right)\right)\;/PGL_{2}\;\left(F_{q}\left[\;\left[t^{-1}\right]\;\right]\right)$ is also given. Moreover, we conclude that the entropy of $\phi_{a}$ with respect to the Haar measure *m*, which is the measure of maximal entropy, is logq.

## Data Availability

Data sharing is not applicable to this article.
